# Computational Fluid Dynamics to Investigate the Possible Behavior of *Ercaicunia multinodosa* While Swimming Inverted on the Water Surface

**DOI:** 10.3390/biology15151252

**Published:** 2026-07-30

**Authors:** Yuhan Li, Zhizhe Yang, Zupeng Zhou

**Affiliations:** 1 School of Mechanical and Electrical Engineering, Guilin University of Electronic Technology, Guilin 541004, China; liyuhan038@163.com (Y.L.); m13145342209@163.com (Z.Y.); 2 Postdoctoral Research Station in Information and Communication Engineering, Guilin University of Electronic Technology, Guilin 541004, China

**Keywords:** *Ercaicunia multinodosa*, swimming inverted, computational fluid dynamics

## Abstract

Inverted swimming behavior has been documented in marine arthropods from the Cambrian to modern oceans. It was previously theorized that this behavior contributed to bringing the center of gravity to a lower position, thereby enhancing balance and stability, and preventing clogging of the mouth in deposit and suspension feeders. In this study, by using the computational fluid dynamics analysis method and wave impact experiments, we evaluate the hydrodynamics of inverted swimming behavior in the early Cambrian *Ercaicunia multinodosa* when it was at the water surface. The results show that when *Ercaicunia multinodosa* swam at the water surface, the inverted swimming was more stable than the normal posture regardless of the direction from which the waves came. Moreover, as the wave amplitude increased (within the tested and validated range of 0.33 to 1.0 times the body height), the rate at which the flipping occurred in the normal posture was accelerated and then remained stable in the inverted posture. Finally, we used a stereolithography apparatus to create a physical model of *Ercaicunia multinodosa* and performed wave impact experiments to verify the research results. Our findings support the hypothesis that the inverted swimming behavior of *Ercaicunia multinodosa* helps increase balance and stability.

## Simple Summary

We hypothesized that the early Cambrian creature *Ercaicunia multinodosa* might have swum upside down at the water surface to stay balanced, after observing similar behavior in some living animals. To test this guess, we used computer simulations and built physical models, then compared how stable the animal was when swimming right-side up versus upside down under waves from different directions. The results showed that the inverted posture remained much more stable than the normal posture in all wave conditions. As waves grew stronger, the right-side-up posture became increasingly likely to tip over, while the upside-down posture stayed steady. These findings support our initial idea that this ancient animal used inverted swimming to maintain stability, much like some modern surface-feeding species. This study offers a clearer picture of how early marine life may have coped with wave action, deepening our understanding of ancient ocean ecosystems and the clever survival strategies that evolved hundreds of millions of years ago.

## 1. Introduction

The study of Cambrian fossils, especially those from the Yunnan Chengjiang Biota deposits, is crucial for understanding the origin and functional morphology of the major arthropod groups [1,2,3,4]. Many details of arthropod morphology have also been uncovered based on high-precision X-ray computed tomography (micro-CT techniques) [5,6,7,8]. However, the influence of unique morphological features on swimming behavior, as well as the foraging behavior of arthropods, has hardly been discussed in studies of their three-dimensional morphological reconstructions. Elucidating the behavior of ancient organisms is a key focus of paleontological research, but this work is largely limited to functional morphology assumptions, lacking reliable computational data to provide evidence [9,10,11].

Previously, Lochhead [12] proposed four factors that may affect swimming direction, namely the aeration of the water, gravity, the surface resistance of the animal, and light. As early as 1936, Foxon [13] and Lowndes [14] provided a reasonable explanation for the inverted swimming posture that is unique to Anostraca and Notostraca. When studying the respiratory function of appendages, they believed that the swimming posture was related to breathing, because in the inverted swimming posture, the appendages can touch the uppermost and best-aerated water layer. In addition, there is another viewpoint that the inverted swimming posture helps to keep the center of gravity to a lower position than in the usual orientation, thereby improving balance and stability [15]. From the perspective of hydrodynamic characteristics, for another inverted swimming arthropod, *Odaraia*, only when swimming upside down does the hydrodynamic characteristics of the carapace help improve performance [16]. Similarly, this may also be the case for *Fibulacaris* [17], but a model of the hydrodynamic characteristics of the carapace is needed to verify this.

When only considering horizontal swimming or floating, the influence of surface resistance on the dorsal and ventral directions is very small [18]. Surface resistance is a particularly important factor in the Cladocera, as they have regular resting pauses during swimming [12]. In addition, the influence of light on the dorsal and ventral directions of Anostraca and Notostraca may be very significant. When illuminated from below, Apus easily swims on its back, which is because, like Anostraca, it may be in a stable position relative to the center of gravity. Inverted swimming is rare in arthropods, mainly limited to the aforementioned Anostraca, Xiphosura [19], some cladocerans [20], and in some cases, Notostraca [21].

Our research team has been collaborating with the Yunnan Key Laboratory for Paleobiology for a long time. In the early stage, we conducted three-dimensional reconstruction research on the fossils of *Ercaicunia multinodosa* provided by the Yunnan Key Laboratory for Paleobiology and obtained a three-dimensional engineering reconstruction model of *Ercaicunia multinodosa* [22]. Analyzing the morphological characteristics of *Ercaicunia multinodosa*, we hypothesized that *Ercaicunia multinodosa* may have exhibited inverted swimming behavior at the water surface, with the functional hypothesis of better access to the well-aerated surface water, facilitating the collection of suspended particles from the water surface, similar to the lifestyle exhibited by *Triops* spp. In order to verify this hypothesis, we used computational fluid dynamics (CFD) to quantify the roll angle, pitch angle, and normal displacement of the two postures under the action of incoming waves in different directions based on a three-dimensional numerical model of *Ercaicunia multinodosa*. The results show that when *Ercaicunia multinodosa* swam at the water surface, the inverted swimming was more stable than the normal posture regardless of the direction from which the waves came. Moreover, as the wave amplitude increases (within the tested and validated range of 0.33 to 1.0 times the body height), the rate at which flipping occurs in the normal posture is accelerated and then remains stable in the inverted posture. Finally, we used the stereolithography apparatus (SLA, Shanghai Liantai Technology Co., Ltd., Shanghai, China) to create a physical model of *Ercaicunia multinodosa* and performed wave impact experiments to verify the research results.

## 2. Materials and Methods

### 2.1. Three-Dimensional Modelling

**Figure 1:**
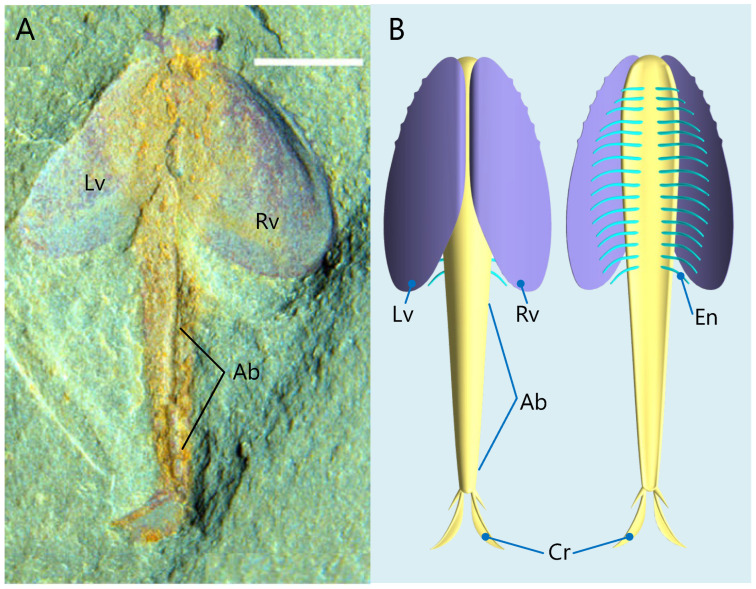
*Ercaicunia multinodosa*, Specimen YKLP 16201, from the Early Cambrian Chengjiang Lagerstätte, South China. (**A**) Dorsal view under light microscope. (**B**) Composite micro-CT model combining the part and counterpart. Lv, left valve; Rv, right valve; En, detail of trunk appendages; Ab, abdominal segments; Cr, caudal ramus; scale bars represent 1 mm.

The fossil material (YKLP 16201) [23] used in this study was from the Yunnan Key Laboratory for Paleobiology, Yunnan University (Figure 1A). Through the computer-aided design program Unigraphics NX (v. 12.0), we constructed an idealized three-dimensional digital model of *Ercaicunia multinodosa* (Figure 1B). The final model is 11 mm long, 3.6 mm wide, and 1.6 mm high.

### 2.2. Computational Fluid Dynamics

**Figure 2:**
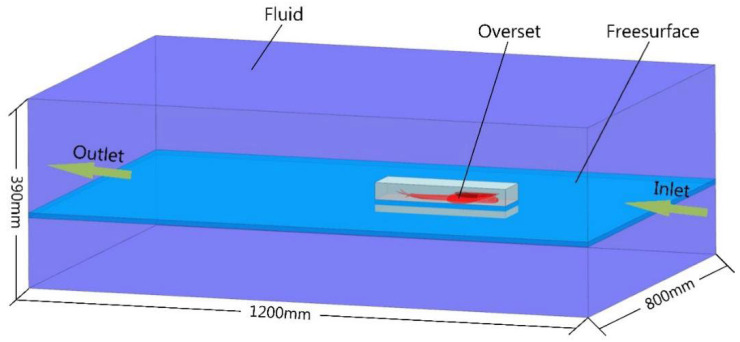
Computational domain used in CFD simulations.

CFD simulations were carried out in Simcenter STAR-CCM+ (15.06.007). The computational domain consisted of a three-dimensional rectangle. First, as shown in Figure 2, we created a three-dimensional rectangular computational domain “Fluid” with a length of 1200 mm, a width of 800 mm, and a height of 390 mm, which represents the completion of the whole simulation process in this region. Then, a “Freesurface” free surface was created for mesh refinement to accurately capture waves. Next, an ‘Overlay’ calculation domain—i.e., the overset mesh region that moves relative to the background mesh—was created to wrap the geometric model of *Ercaicunia multinodosa* and simulate its movement. Then, a Boolean operation was used to subtract the *Ercaicunia multinodosa* model from the “Overlay” calculation field. The “Transforms” command was used to set the calculation field to “positive floating” or “inverted swimming”.

**Figure 3:**
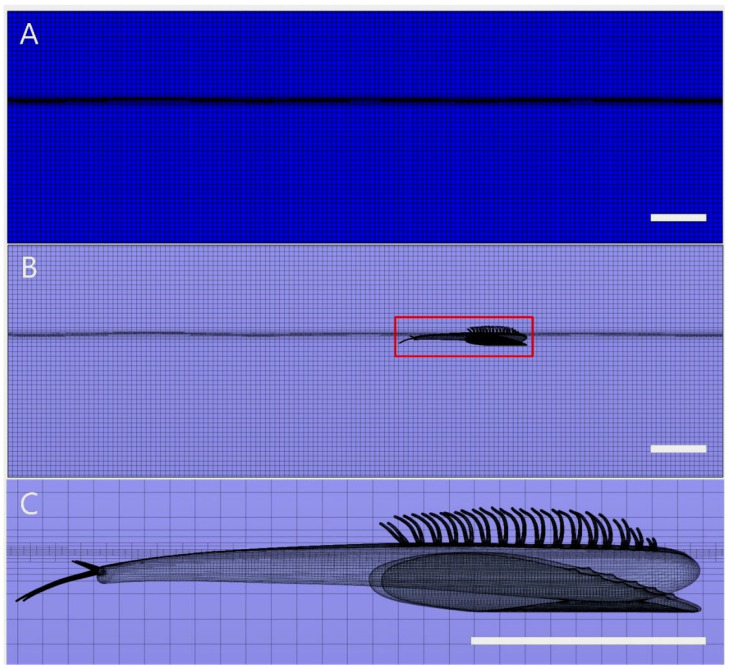
Mesh used in CFD simulations. (**A**) Mesh in the computational domain. (**B**) Detail of the mesh near *Ercaicunia multinodosa*. (**C**) Detail of the mesh within the body of influence. Scale bars represent 5 cm (**A**); 3 cm (**B**); 0.3 cm (**C**).

An automatic mesh was created for the “Overlay” area. To better capture the geometric complexity of the *Ercaicunia multinodosa* model, an unstructured mesh was used. The base size is 20 mm, the minimum surface size was 2, the number of primary layers was 6 layers, and the maximum cell size was 10. Then, a “Surface Control” was created to mesh the surface of *Ercaicunia multinodosa* (the red part in Figure 2) and the “overlay” boundary surface. The target surface size was 10% of the base size. Finally, the grid control was applied for the fluid area, and the customized anisotropic size was selected in the Z direction corresponding to the wave height, in order to improve calculation accuracy under the action of waves. The final grid consists of 8.47 million units (Figure 3).

To quantify the numerical uncertainty, a grid convergence study based on the Grid Convergence Index (GCI) method was performed. Three systematically refined meshes were generated with a constant refinement ratio of *r* = $\sqrt{2}$, resulting in approximately 4.23 million (coarse), 5.99 million (medium), and 8.47 million (fine) cells. The flip time—the key output parameter for our stability assessment—was monitored across the three meshes, yielding values of approximately 5.0 s (coarse), 4.2 s (medium), and 4.0 s (fine). Following the recommended GCI procedure, the apparent order of convergence *p* was calculated as approximately 4.0, and the GCI for the fine mesh was determined to be 2.08%. The relatively high apparent order of convergence (*p* ≈ 4.0) is attributed to the threshold nature of the flip time—a nonlinear integral response to the wave-induced motion. Such quantities can exhibit convergence rates that differ from the nominal spatial order of the underlying discretization. This value is well below the generally accepted threshold of 5%, confirming that the numerical uncertainty associated with grid resolution is within an acceptable range and that the current 8.47-million-cell mesh provides grid-converged results for the key flipping behavior analyzed in this study.

Having established numerical convergence through the GCI analysis above, we further examined our grid resolution against the ITTC-recommended practice for wave simulations. The ITTC guidelines suggest a horizontal grid spacing of *λ*/80 (5 mm in our case, as *λ* = 400 mm) and a vertical grid spacing of *Hw*/20 for accurate wave propagation. In our simulations, the base horizontal grid size was 20 mm—coarser than the strict ITTC recommendation—because our primary concern is the rigid-body flipping response (roll and heave) rather than the precise evolution of the waveform. In the vertical direction, however, anisotropic refinement was applied near the free surface, yielding a minimum grid spacing of approximately 0.1 mm in the Z-direction. We acknowledge that for the smallest tested wave height (0.33 mm), this vertical spacing does not fully satisfy the *Hw*/20 criterion (which requires 0.0165 mm).

Nevertheless, the adequacy of this resolution is justified by two considerations. First, the GCI_fine value of 2.08% confirms that the numerical uncertainty for the key output parameter—flip time—is well within the acceptable limit of 5%. Second, the qualitative consistency between the CFD predictions and the physical wave-tank experiments provides independent empirical support for the simulated flipping trends. Therefore, while we acknowledge the deviation from the strict ITTC grid guidelines, we are confident that the current mesh resolution is sufficient to capture the dominant stability-related motion of *Ercaicunia multinodosa* and does not compromise the main conclusions of this study.

**Figure 4:**
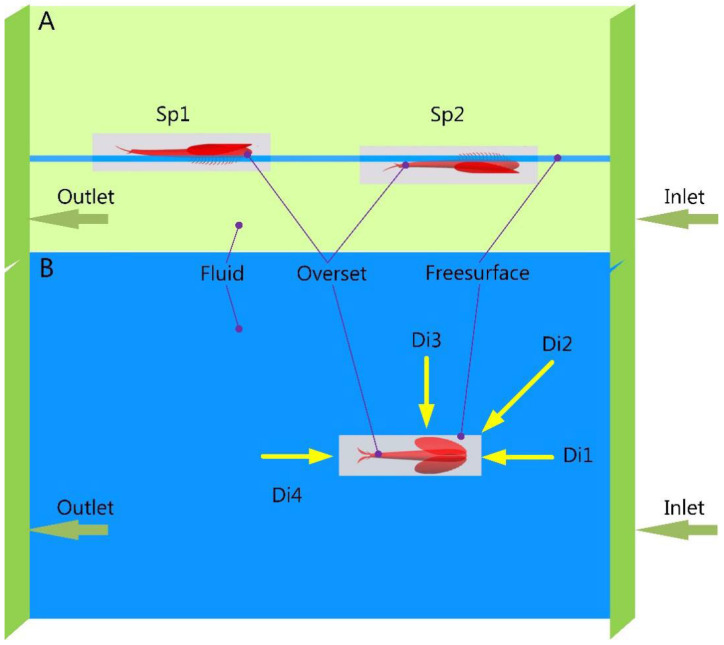
Region of influence within the computational domain. (**A**) Front view of the computational domain. (**B**) Vertical view of the computational domain. Sp1, the initial state is normal swimming. Sp2, the initial posture is inverted swimming. Di1~Di4 represent four different wave flow directions, namely forward, oblique, vertical, and backward.

Next, create a “physics continuum” with the attribute set to “Multiphase”, one of which is “liquid” and the other is “Gas”. Create a “First Order of Wave” with an advanced direction of [−1.0, 0.0, 0.0], indicating that the wave flows from the head to the tail of *Ercaicunia multinodosa* (Di3 in Figure 4), with an amplitude of 1 mm and a wave length of 400 mm. Next, define the boundary conditions for the computational domain. The flow velocity at the front of the computational domain is defined as the velocity inlet boundary, and the flow velocity at the rear of the computational domain is defined as the outflow boundary. The velocity function is the “Velocity of First Order VOF Wave”. The top and sides of the computational domain are defined as sliding and symmetric boundary conditions, respectively, and no-slip boundary conditions are specified for the surface of the *Ercaicunia multinodosa* model and the lower surface of the domain.

In addition to the baseline wave condition (amplitude 1 mm, wavelength 400 mm) used to set up the computational domain and physics, five wave-amplitude conditions were tested to evaluate the effect of wave amplitude on flipping behavior. These conditions were defined as 0.2×, 0.33×, 0.66×, 1.0×, and 1.33× the body height of *Ercaicunia multinodosa* (body height = 1.6 mm), corresponding to wave amplitudes of 0.32, 0.528, 1.056, 1.60, and 2.128 mm, respectively. The multipliers (0.2× to 1.33×) refer to wave amplitude (the maximum displacement of the water surface from the still-water level), not the crest-to-trough wave height. For a simple sinusoidal wave, the crest-to-trough wave height is twice the amplitude. The wavelength was kept constant at 400 mm for all conditions, and all other simulation parameters—including computational domain size, boundary conditions, mesh resolution, and DFBI settings—remained identical across conditions to ensure consistency. The wave was generated using the ‘First Order VOF Wave’ model in STAR-CCM+ with the propagation direction set according to the four inflow directions (Di1–Di4 in Figure 4).

The purpose of the simulation was to observe the stability of *Ercaicunia multinodosa* in the positive and upward floating states, so it was also necessary to define the moving body. Set “DFBI Rotation and Translation” for the overlay area. The degrees of freedom were the rotation around the x-axis and y-axis and translation along the z-axis, corresponding to the rolling, pitching, and undulating heights of *Ercaicunia multinodosa* under the action of waves. Calculate the moment of inertia of the solid model of the *Ercaicunia multinodosa* and input [3.3056 × 10^−6^, 8.3996 × 10^−5^, 8.5504 × 10^−5^] according to the diagonal components.

### 2.3. Wave Impact Experiment

**Figure 5:**
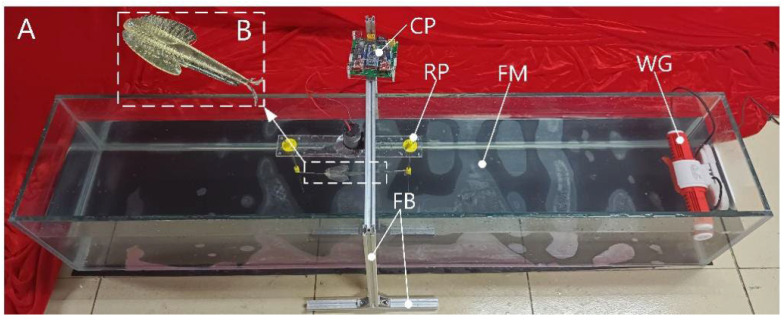
Wave impact experiment on the printed model of *Ercaicunia multinodosa*. (**A**) Wave impact experimental platform. (**B**) *Ercaicunia multinodosa* printed model. CP, control panel. RP, rotating platform. FM, fluid medium. WG, wave-generation devices. FB, fixed bracket.

An experimental platform was established for conducting wave impact tests on *Ercaicunia multinodosa* to verify the results from the CFD simulations. (Figure 5A). Due to limitations in the processing technology of physical models, it is not possible to produce models at the original scale. In order to minimize the fluid mechanics impact of the magnification model, we used an 8-fold magnification model for SLA printing (Figure 5B). The printed *Ercaicunia multinodosa* model was connected to the rotating platform through thin wires, which was rotated by a motor to ensure that the printed model can be impacted by forward waves, oblique waves, vertical waves, and backward waves, which can correspond to the wave inflow direction set in the simulation conditions. Select water as the fluid medium and use wave-generation devices to create different forms of waves. Then, conduct impact experiments on *Ercaicunia multinodosa* printed model with different directions and amplitudes of wave inflow.

It should be noted that the physical model was printed at 8× magnification due to the resolution limitations of the SLA printer. Strictly speaking, this scaled-up model does not simultaneously preserve all dimensionless numbers (Reynolds, Froude, and Weber numbers) that govern the air–water interfacial hydrodynamics. However, the physical experiments in this study were designed for qualitative validation rather than quantitative calibration. They were used to verify the directional trend of flipping behavior (i.e., whether the normal posture flips while the inverted posture remains stable, and the relative order of wave-direction susceptibility) predicted by the CFD simulations. The consistency between the experimental observations and the CFD predictions—particularly the flipping sequence (vertical > oblique > forward > backward)—supports the validity of the CFD setup. We acknowledge that the use of a magnified model is a limitation in terms of exact dynamic similarity, and future work using micro-scale experimental techniques or full numerical approaches could further refine this aspect.

## 3. Results

**Figure 6:**
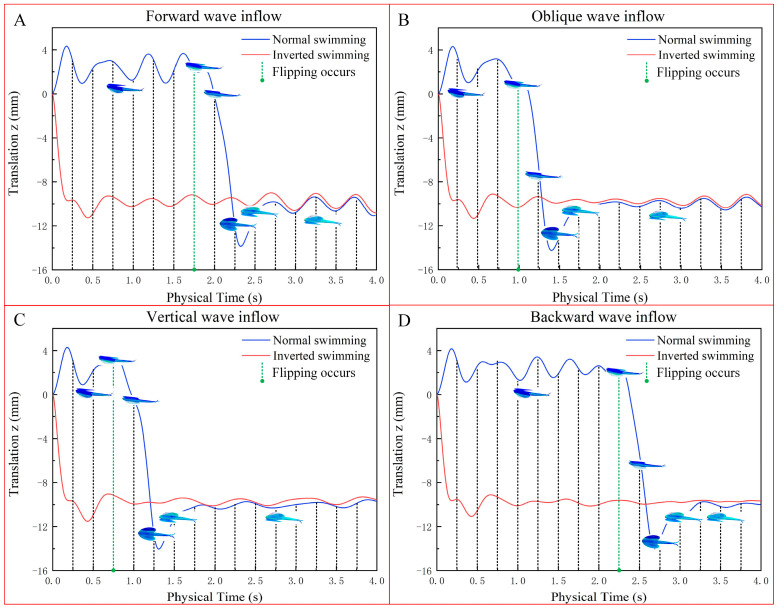
Under the action of four different directions of waves and currents, the initial postures of *Ercaicunia multinodosa* are inverted swimming and normal swimming, respectively. (**A**–**D**) correspond to forward waves, oblique waves, vertical waves, and backward waves inflow, respectively. The x-axis represents the simulation time, and the y-axis represents the height of the center of gravity. The blue and red curves represent the changes in the center of gravity during normal swimming and inverted swimming, respectively, while the green dashed line represents the time when flipping occurs.

We set up four different directions of wave flow, forward, oblique, vertical, and backward. The simulation results show (Figure 6) that under the action of four different wave directions, when the initial posture of *Ercaicunia multinodosa* is an inverted swimming posture, no flipping occurs. When the initial posture of *Ercaicunia multinodosa* is the normal swimming position, it flips over. From Figure 6, it can also be seen that the direction of wave flow is different, and the time of overturning also varies. The vertical wave inflow causes overturning first, followed by oblique wave inflow, then forward wave inflow; backward wave flow causes overturning last. The negative displacement of the center of gravity shown in Figure 6 does not indicate that *Ercaicunia multinodosa* sank below the free surface. Rather, it reflects the vertical oscillatory motion of the body in response to wave forcing, with the reference frame set at the initial center-of-gravity position. The negative values correspond to downward heave motion during the wave trough, while positive values correspond to upward motion at the wave crest. Throughout the simulation, the body remained at the air–water interface and did not submerge permanently. This oscillatory behavior is consistent with the expected heave response of a floating body under wave action.

**Figure 7:**
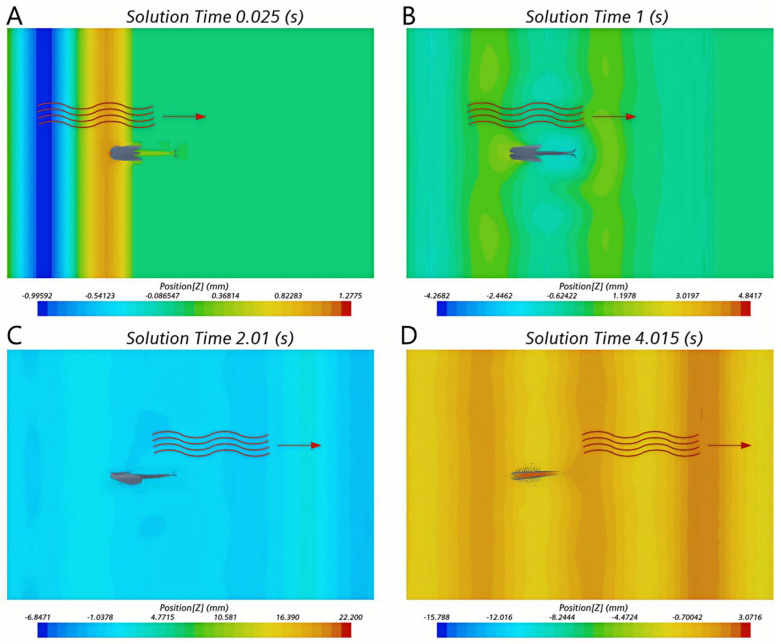
The flipping process of *Ercaicunia multinodosa* under the action of forward waves. From (**A**–**D**), *Ercaicunia multinodosa* flipped 180° under the action of forward waves.

We conducted post-processing on the simulation calculation results, as shown in Figure 7, which shows the flipping process of *Ercaicunia multinodosa* under the action of forward waves when the initial posture is normal swimming. We also obtained a post-processed simulation video (Video S1), from which we can clearly see the flipping situation of *Ercaicunia multinodosa* under the action of waves.

**Figure 8:**
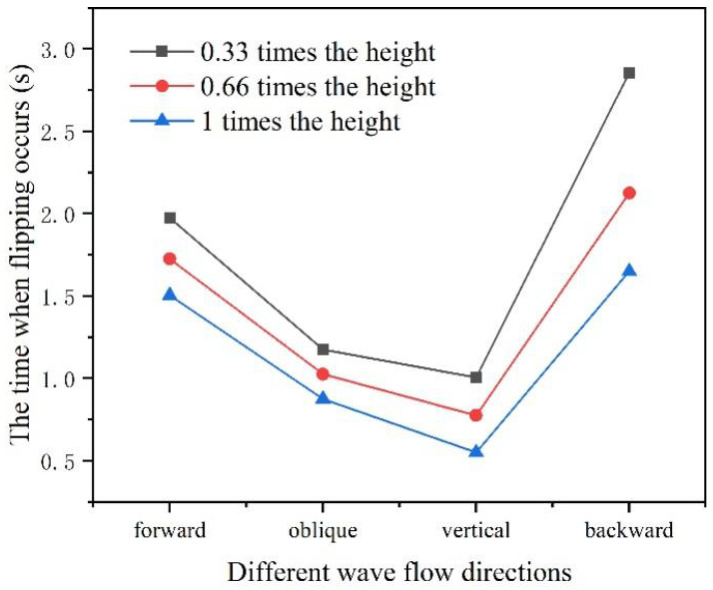
Effect of different wave amplitudes on the flipping of the normal swimming posture of *Ercaicunia multinodosa*. The horizontal axis represents the four different wave inflow directions (forward, oblique, vertical, and backward); the vertical axis represents the time at which flipping occurs. Three wave-amplitude conditions are shown: 0.33×, 0.66×, and 1.0× the body height. The 0.2× wave amplitude condition was excluded because the wave amplitude was too small to trigger flipping, and the 1.33× wave amplitude condition was excluded because the excessive wave amplitude washed the organism out of the computational domain, resulting in a numerical error rather than a valid physical result. The data shown are from CFD simulations; the physical experiments (Supplementary Video S2) qualitatively confirmed the predicted flipping trends.

In order to study the effect of different wave amplitudes on the flipping of the normal swimming posture of *Ercaicunia multinodosa*, we set five different wave amplitudes, namely 0.2 times, 0.33 times, 0.66 times, 1 time, and 1.33 times the height of *Ercaicunia multinodosa* itself, and conducted CFD simulation calculations. The simulation results show (Figure 8) that when the wave amplitude is 0.2 times the body height, the amplitude is too small to trigger flipping. When the wave amplitude is 1.33 times its own height, the excessive wave amplitude causes *Ercaicunia multinodosa* to be washed out of the computational domain, resulting in a numerical error rather than a valid physical result. These two conditions are therefore excluded from the valid data range. Within the valid range of 0.33 to 1.0 times the body height, *Ercaicunia multinodosa* remains stable when the initial posture is inverted swimming, whereas when the initial posture is normal swimming, flipping occurs; moreover, the larger the wave amplitude, the faster the flipping occurs (Figure 8).

We filmed the whole process of wave impact experiments on the printed model of *Ercaicunia multinodosa* and made a video (Video S2). From the video, it can be seen that when the initial posture of the *Ercaicunia multinodosa* printed model is normal swimming, it flips under the action of waves, and then stabilizes in an inverted swimming posture. Subsequently, under the action of vertical waves, the first flip occurs, followed by oblique waves, then forward waves, and finally backward waves. The experimental results are consistent with the simulation results, confirming our hypothesis that *Ercaicunia multinodosa* adopts an inverted swimming posture at the water surface.

It should be noted that the wave impact experiments were repeated at least three times for each combination of wave direction and wave amplitude to assess repeatability. Across all replicates, the flipping behavior was consistently reproduced: the normal posture invariably flipped under wave action while the inverted posture remained stable, and the relative flipping sequence (vertical > oblique > forward > backward) was observed without exception in every trial. Therefore, the results presented above are representative of these repeated trials, confirming that the experimental observations are highly repeatable. Supplementary Video S2 provides a continuous, unedited recording of a representative trial, which illustrates the typical flipping behavior observed across all replicates. The repeated trials were recorded to confirm qualitative consistency; frame-by-frame timing analysis was not performed. All replicates showed the same directional sequence (vertical > oblique > forward > backward) and the same flipping occurrence without exception, confirming full repeatability at the qualitative level. Future work incorporating high-speed video and quantitative timing analysis could further substantiate these findings.

## 4. Discussion

The Cambrian arthropod *Ercaicunia multinodosa* was described in 1999 [24] and has since been mentioned several times [25]. This remained the case until Zhai and colleagues [23] used X-ray three-dimensional imaging technology (micro-CT) with a precision of micrometers to observe 20 pairs of well-preserved appendages and their amazing morphological details of *Ercaicunia multinodosa*. In previous studies [22], we focused on the reconstruction of the compressed and missing parts of *Ercaicunia multinodosa* fossils from an engineering perspective, and obtained its three-dimensional reconstruction model. Upon observing the reconstructed model of *Ercaicunia multinodosa*, we found that its appearance is very similar to that of the extant analogues *Triops* spp., which exhibit an ‘interesting’ behavior of often swimming in an inverted posture at the water surface (Video S3). From this, we speculate that this behavior may also have existed in the early Cambrian and used CFD simulation calculation and experiments to provide strong evidence for this speculation.

Currently, there is very little research on the fluid dynamics characteristics of air–water interfaces and their biological significance, as this is a multidisciplinary issue. Swimming at or near the air–water interface (i.e., without bottom interaction) increases the difficulty of maintaining stability due to the dispersion of surface waves, compared to swimming in deep water [26,27,28]. The drag of a rigid body swimming at a constant speed downstream of the water surface is approximately five times that of swimming in deep water [26]. In addition to friction and pressure drag, wave forces can be a major factor affecting organisms swimming near the air–water interface. The wave pattern is generated by two moving pressure points, one located downstream of the organism and the other upstream. The interference between these two wave systems is determined by the relationship between velocity and length. At certain speeds, lateral wave peaks from the head of the organism may combine with lateral wave peaks from the tail of the organism to produce large wave trains, while at other speeds, the wave systems from the head and tail cancel each other out, producing small wave trains [29]. Both large and small wave trains can cause instability in the swimming of organisms at the water surface, similar to the roll, pitch, and heave phenomena that occur during ship operation. The scale effect associated with the 8× magnified physical model, while limiting quantitative comparisons, does not affect the qualitative validation of the predicted flipping trends.

**Figure 9:**
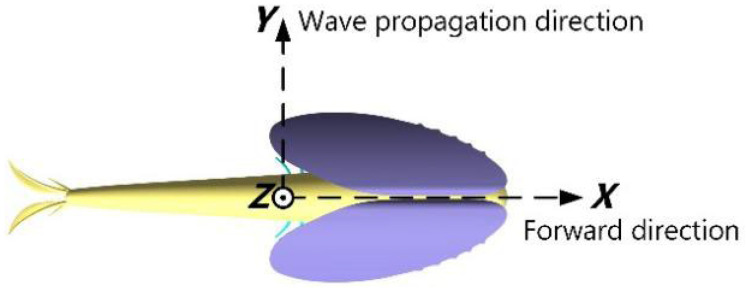
Coordinate system.

The global stability analysis method for ships in random waves can be used to calculate the stability of *Ercaicunia multinodosa* when it is subjected to wave action at the water surface. In the study of the stability of *Ercaicunia multinodosa*, we start from a two-dimensional problem and focus on the transverse and vertical rocking motion of *Ercaicunia multinodosa*. Firstly, we establish a motion coordinate system (Figure 9).

Consider the motion of *Ercaicunia multinodosa* as a coupling of the transverse and vertical rocking motions. The stability height can be expressed by the following equation:

*G_M_* = *G*_*M*0_ + ∆*G*_*M*1_ (1)

*G_M_*_0_ is the stability height of *Ercaicunia multinodosa* during roll motion, and ∆*G_M_*_1_ is the change in stability height during roll motion. According to the Taylor formula, when the draught of *Ercaicunia multinodosa* changes, the stability height is calculated as follows:

(2) $$G_{M}=G_{M0}+\frac{\partial G_{M}}{\partial z}z+\frac{1}{2}\frac{\partial^{2}G_{M}}{\partial z^{2}}z^{2}+\ldots +\frac{1}{n}\frac{\partial^{n}G_{M}}{\partial z^{n}}z^{n}$$

If the change in stability height after the second-order transverse rocking motion of *Ercaicunia multinodosa* is ignored, the stability height can be obtained as:

(3) $$G_{M}=G_{M0}+\frac{\partial G_{M}}{\partial z}z$$

By solving the equation, it can be concluded that

(4) $$G_{M}=G_{M0}+\left[\frac{\partial KB}{\partial T}+\frac{\partial \left[I_{g}\right]}{\partial T}\right]Z$$

In the formula: *KB* is the height of the floating center of *Ercaicunia multinodosa*, *T* is the draft depth, and *Z* is the center of mass of *Ercaicunia multinodosa*.

Under the action of wave loads, the height of the floating center of *Ercaicunia multinodosa* continuously changes. Combining this with the Euler equation, the equation for the height of the floating center of *Ercaicunia multinodosa* can be obtained as follows:

(5) $$H_{0}=A_{w}{\left(\frac{Z}{T}\right)}^{n}$$

In the formula, *A_w_* is the area of wave action on the body of *Ercaicunia multinodosa*, and n is the wave height coefficient.

According to Formula (5), it can be concluded that under the action of waves, the variation of the floating center height of *Ercaicunia multinodosa* is directly proportional to the impact area *A_w_*, directly proportional to the height of the center of mass *Z*, and inversely proportional to the draft depth *T*. Moreover, the larger the wave height, the more intense the floating change.

The CFD simulation results presented in the previous section show that under the same wave action, the inverted swimming posture is more stable than the normal swimming posture. It can be explained in Formula (5) that inverted swimming can reduce the height of the center of mass and increase the draft depth. When the wave direction is different, the vertical wave flow first causes flipping, followed by the oblique wave flow, then the forward wave flow, and finally the backward wave flow causes flipping. This can be explained by Formula (5) that when the vertical wave propagates, the area of *Ercaicunia multinodosa* receiving the wave impact is the largest, and when the backward wave propagates, the area of *Ercaicunia multinodosa* receiving the wave impact is the smallest. Within the normal wave height range, the larger the wave height, the faster the flipping occurs. It can be explained in Formula (5) that the larger the wave height, the greater the coefficient n, resulting in a more drastic change in the height of the center of buoyancy.

While our results support the stability-enhancement hypothesis for inverted swimming in *Ercaicunia multinodosa*, alternative functional interpretations should be considered. First, predator avoidance—whereby an inverted posture near the surface reduces visual contrast against the bright water surface for predators below—has been proposed for some modern aquatic invertebrates [20]. Second, phototaxis, or positive phototactic orientation toward light from above, could also contribute to inverted positioning [12]. Third, the inverted posture may be a consequence of appendage and feeding morphology rather than a direct adaptation for stability: if the primary feeding appendages are ventral in orientation, inverted swimming would position them optimally for capturing suspended particles at the air–water interface. However, our hydrodynamic analysis demonstrates that even without considering feeding function, the inverted posture is significantly more stable than the normal posture under wave action. This suggests that even if inverted swimming originated as a byproduct of morphological or feeding constraints, the stability advantage it confers would constitute an independent selective pressure favoring its maintenance. These explanations are not mutually exclusive with the stability hypothesis, and it is plausible that multiple selective pressures acted in concert. Future studies combining CFD with behavioral ecology modeling could help disentangle these factors.

If regarded as capable of swimming on the surface of the water, *Ercaicunia multinodosa* is likely to swim upside down, similar to *Triops* spp. This behavior is supported by the hydrodynamic properties of its external contour. The inverted swimming lifestyle has been identified in the Cambrian arthropods *Odaraia* [30] and *Sarotrocercus* [31], and functional morphology has been inferred. *Fibulacaris* may also have exhibited this behavior. In addition to *Triops*, inverted swimming has also been documented in *Xiphosurida* [19], which shares a marine habitat with *Ercaicunia multinodosa*. Although horseshoe crabs differ substantially in body plan—lacking the bivalved carapace characteristic of *Ercaicunia multinodosa*—their inverted swimming behavior offers an interesting parallel in terms of ecological context. Future comparative studies between morphologically disparate groups may further elucidate the hydrodynamic principles underlying convergent inverted swimming adaptations.

We can conclude that inverted swimming helps to lower the center of gravity, thereby improving balance and stability. For suspended feeders, inverted swimming can avoid sediment clogging their feeders. As a matter of fact, the above inference requires fluid dynamics modeling of the hydrodynamic properties of organisms or crustaceans to verify. The computational fluid dynamics analysis used in this article can provide a verification method for functional morphology studies of other paleontological organisms. Based on the overall morphology and potential suspension-feeding habitat, *Ercaicunia multinodosa* may have swum inverted, probably while at the water surface, as inferred in morphologically similar species.

## 5. Conclusions

Herein, we used computational fluid dynamics simulations to investigate whether the early Cambrian arthropod *Ercaicunia multinodosa* appeared in an inverted swimming posture at the water surface. We made a physical model and verified the simulation results through wave impact experiments. Our findings support the hypothesis that the inverted swimming behavior of *Ercaicunia multinodosa* helps increase balance and stability. Modeling hydrodynamic properties can provide quantitative data support to validate the hypotheses of functional morphology. This work opens new perspectives for the study of paleontology.

## Data Availability

Data are contained within the article and Supplementary Materials.

## Supplementary Materials

The following supporting information can be downloaded at: https://www.mdpi.com/article/10.3390/biology15151252/s1. Video S1: Demonstration video of the computational fluid dynamics simulation process; Video S2: The whole process of wave impact experiments on the printed model of *Ercaicunia multinodosa*; Video S3: Recording of the inverted swimming phenomenon in *Triops*.
